# Enhanced Photo-Assisted Acetone Gas Sensor and Efficient Photocatalytic Degradation Using Fe-Doped Hexagonal and Monoclinic WO_3_ Phase−Junction

**DOI:** 10.3390/nano10020398

**Published:** 2020-02-24

**Authors:** Ji-Chao Wang, Weina Shi, Xue-Qin Sun, Fang-Yan Wu, Yu Li, Yuxia Hou

**Affiliations:** 1 College of Chemistry and Chemical Engineering, Henan Institute of Science and Technology, Xinxiang 453000, China; wangjichao@hist.edu.cn (J.-C.W.); Sxq13683887416@163.com (X.-Q.S.); wu18568573582@126.com (F.-Y.W.); 2 College of Chemistry and Molecular Engineering, Zhengzhou University, Zhengzhou 450052, China; 3 College of Chemistry and Chemical Engineering, Xinxiang University, Xinxiang 453000, China; 4 College of Chemical Engineering and Materials, Zhengzhou University of Light Industry, Zhengzhou 450000, China; 2016030@zzuli.edu.cn

**Keywords:** photo-assisted, gas sensor, acetone, Fe doping, phase−junction

## Abstract

The development of WO_3_-based gas sensors for analysis of acetone in exhaled breath is significant for noninvasive diagnosis of diabetes. A series of Fe-doped hexagonal and monoclinic WO_3_ phase−junction (Fe−*h/m*−WO_3_) sensors were synthesized by the hydrothermal calcination method, and the influences of operating temperature and light irradiation on the response were studied. Under light emitting diode (LED) illumination, Fe−*h/m*−WO_3_ exhibited higher responses to acetone than those of the undoped WO_3_-based sensors at an operating temperature of 260 °C with 90% relative humidity, and good linearity between response and acetone concentration (0.5 to 2.5 ppm) was achieved under the 90% relative humidity condition. Meanwhile, the optimal Fe−*h/m*−WO_3_ sensor exhibited high selectivity and stability for a duration of three months. The excellent sensing performance of Fe−*h/m*−WO_3_ was attributed to the formation of phase−junction and Fe doping, and these were beneficial for the separation of photon−generated carriers and oxygen adsorption on the WO_3_ surface, promoting the generation of superoxide radicals, which was demonstrated by electron paramagnetic resonance and photocurrent tests. Additionally, the Fe−doped WO_3_ phase−junction sample also showed good photocatalytic performance for rhodamine B degradation. This study may provide some insights into rational design of new types of gas sensors and offer an alternative for noninvasive diagnosis of diabetes.

## 1. Introduction

Over the last few years, breath analysis, as a rapid, cheap and non−invasive biological method, has attracted much attention in diagnosing and monitoring medical fields [1,2,3]. Among the multiple inorganic gases (e.g., O_2_, CO_2_, NO, NH_3_) and volatile organic compounds in the exhaled breath, acetone, as a generally accepted biomarker for type−I and type−II diabetes, has been studied in detail and used to monitor metabolic disorders [4,5]. The analysis of acetone in the exhaled breath exhibits higher sensitivity than the detection of blood glucose [6]. The clinical data indicated that the exhaled acetone of healthy people was believed to be lower than 0.9 ppm, while that of diabetes exceeded 1.8 ppm [7,8,9]. It is meaningful to explore low−cost and steady gas sensors for detecting low concentrations of acetone in the exhaled breath.

Up to now, semiconductor gas sensors have been widely known for their excellent response, superior testing precision, low cost and long working life [3,10]. In a typical semiconducting sensor head, metal oxides including ZnO [11], TiO_2_ [12], MoS_2_ [13] and NiFe_2_O_4_ [4] have been widely admitted as good sensing materials for multiple gases, due to the change of electric conductivity, caused by the interaction of gas molecules and sensing material. Tungsten trioxide (WO_3_), as a n−type semiconductor, has been extensively used as a gas sensor material for detecting toxic gases such as NO_2_, NH_3_, H_2_S and acetone [13,14,15]. However, the pure WO_3_ material is still unable to meet the current demand for low concentration acetone detection limits and high selectivity in breath analysis [1,2]. Various approaches, including regulating particle size, controlling morphology, designing crystal structure, building surface defects and adjusting interface properties, have been adopted to solve the above problems [10,16,17]. Li et al. [18] reported that the gas-sensing property of a Co-doped monoclinic phase WO_3_ for acetone molecules was significantly improved due to the effect of lattice defect and Co doping, which facilitated gas adsorption. It is noteworthy that various band structures and interface properties existed in different WO_3_ materials [19,20]. In the lattice structure of WO_3_ with a hexagonal phase, the open structure could affect the adsorption property on the surface [21]. Additionally, the separation of photon-generated carriers is vital for the sensitivity of metal oxide based gas sensors [10]. The construction of semiconductor phase-junctions becomes one of the effective approaches for enhanced carrier separation. Li et al. [22,23] reported that the anatase−rutile phase junction of TiO_2_ greatly enhanced the photocatalytic activity, and then they demonstrated that efficient charge separation and transfer were achieved across the α−β phase junction of Ga_2_O_3_ leading to enhanced photocatalytic performance. Hence, the formation of WO_3_ phase junctions with hexagonal and monoclinic phase could promote the electron hole separation.

Apart from the carrier separation in semiconductors, the acetone-sensing property of the semiconductors relies heavily on the surface conductivity induced by chemical reactions between the target gases and oxygen species adsorbed onto the surface [17]. As for the general acetone-sensing mechanism (Equations (1)–(4)) [18], the oxygen ions were formed by drawing electrons from the conduction band of metal oxide, and different oxygen species (O_2_^−^, O^−^ and O^2−^) were formed depending on the operating temperature [16].

O_2_(ads) + e^−^ → O_2_^−^·(ads) (1)

O_2_^−^·(ads) + e^−^ → 2 O^−^·(ads) (2)

O^−^·(ads) + e^−^ → O^2−^·(ads) (3)

CH_3_COCH_3_(gas) + O^2−^ → CO_2_ + H_2_O + 2e^−^ (4)

Based on the mechanism, the enhancement of oxygen adsorption ability was a positive approach for increasing gas-sensing performance of semiconductors. Sukunta et. al. found that Fe(III)-doping in SnO_2_ could result in the creation of holes through the defect reactions, leading to the change of atoms surrounding the adsorption sites and further resulting in a large change of conductivity in air and in target gas [24]. Guo et. al. further proved that the oxygen vacancies in Fe-doped ZnO often acts as an adsorption and a reaction site to form plentiful O_2_^−^ and O^2−^, and Fe-doping provided a large number of gas adsorption sites and could trap electrons from ZnO easily, leading to the enhancement of gas sensitivity [25]. Besides, UV−visible light irradiation created a charge transport that increased the density of free electron–hole pairs in WO_3_ semiconductor [26]. The Fe-doping not only could produce oxygen vacancies, promoting the oxygen adsorption ability, but also formed impurity energy levels in the band gap, increasing the light energy utilization [27]. Hence, Fe-doped WO_3_ phase junctions may be an applicable material for photo-assisted exhaled analysis. 

In this study, Fe-doped hexagonal and monoclinic WO_3_ junction was synthesized, and the photocatalytic performance for rhodamine B (RhB) degradation was studied under visible-light illumination. The gas-sensing performances of the prepared samples including sensitivity, selectivity and stability were comprehensively investigated under light emitting diode (LED) light illumination, and the influences of operating temperature and relative humidity (*RH*) on the analysis of acetone content were discussed. The response was improved, and the optimal operating temperature was lower than the pure WO_3_ sensor [28,29,30]. Fe-doped WO_3_ phase junctions exhibited excellent linearity at 90% *RH* atmosphere under LED illumination. The gas-sensing mechanism under illumination was finally proposed.

## 2. Materials and Methods 

### 2.1. Materials Synthesis

A series of Fe-doped hexagonal and monoclinic WO_3_ phase junction samples were synthesized by the solvothermal calcination method. Typically, 0.56 g ammonium metatungstate was dissolved into 30 mL HCl solution (6 mol/L) under magnetic stirring, and then different amounts of Fe(NO_3_)_3_·9H_2_O were added. The obtained reddish solution was subsequently placed into a Teflon−lined autoclave and maintained at 180 °C for 6.5 h. The precursor was collected by centrifugation and washed with ethanol, along with vacuum drying. The final products were obtained by annealing at 475 °C for 2 h and denoted as *x*Fe−*h/m*−WO_3_, where *x* represented the Fe/W molar ratio. For comparison, the undoped WO_3_ phase junction (*h/m*−WO_3_), monoclinic WO_3_ (*m*−WO_3_) and hexagonal WO_3_ (*h*−WO_3_) samples were synthesized by the similar procedure without the existence of Fe(NO_3_) _3_·9H_2_O and annealed at 475, 415 and 500 °C, respectively. The contents of W and Fe were identified by the inductively coupled plasma atomic emission spectrometer (ICP−AES) measurements (Table S1).

### 2.2. Gas Sensor Fabrication

About 15 mg of the WO_3_-based samples were mixed with 2~3 drops of terpineol to form a paste, which was then coated uniformly onto the surface of a ceramic tube with a pair of gold electrodes with a gap length of 2 mm and connected by platinum wires. The obtained WO_3_ sensing film was dried at 60 °C for 2 h. A Ni−Cr resistor wire was put through the ceramic tube as a heater to achieve the required working temperature. All the fabricated WO_3_ gas sensors were aged at 300 °C for 7 days in air.

### 2.3. Structural Characterization

The crystal structures of the obtained samples were determined by powder X−ray diffractometer (PANalytical X’Pert PRO, Netherlands) operating at 40 kV and 35 mA, using Cu Kα radiation (λ =1.5418 Å) with a scanning speed of 0.2 °/s. The surface area was measured by N_2_ absorption−desorption test using automated surface area and prose size analyzer (BELSORP−max, MicrotracBEL Inc., Japan). The morphology of the products was determined with a JEOL JEM-2010 and Tecnai G^2^ F20 (FEI Inc., USA) at the operating voltage of 200 kV. In high-resolution transmission electron microscopy (HRTEM), the corresponding fast Fourier transform (FFT) was obtained by Gatan Digital Micrograph software (Gatan Inc., America). X−ray photoelectron spectroscopy (XPS) measurements were carried out on a X−ray photoelectron spectrometer (PHI−5400, Perkin Elmer Inc., USA). Al *Kα* radiation (*hv* = 1486.6 eV) was adopted as the excitation source and the binding energies were corrected using the background C1s peak (284.6 eV) as a reference.

### 2.4. Performance Measurements

**Figure 1:**
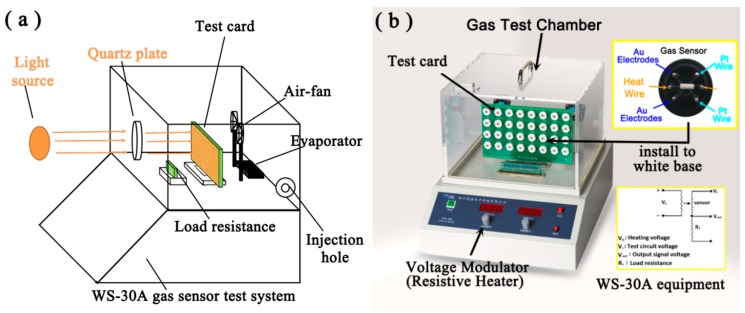
Schematic diagram (**a**) of the light−driven gas sensor test system and photograph (**b**) of original WS−30A equipment.

The gas-sensing properties were measured by the WS-30A gas sensor test system (Zhengzhou Wei Sheng Electronics Technology Co. Ltd., Zhengzhou, China, as shown in Figure 1). The tested gases such as acetone, methanol, ethanol, toluene, CO, NO and NH_3_ were injected into the closed system, respectively. The response and selectivity were assessed in ambient atmosphere at controlled operating temperature (200−360 °C) under various concentrations. The relative humidity (*RH*) in environment was controlled by adding additional water on the evaporator. Before the addition of target analytes, liquid water was firstly injected by the micro-syringe and vaporized immediately on the evaporator. Fast gasification and diffusion of water occurred by the evaporator and air fan in the gas test chamber, and water was added to the chamber until the humidity reached experimental requirements. The electrical resistance of the sensors in air or in the target gas is calculated as

R = [R_L_ / (V_c_ − V_out_)]/V_out_ (5)

where R, R_L_, Vc and V_out_ were the sensor resistance, the load resistance, the total loop voltage applied to the electrical circuit and the output voltage across the load resistor, respectively. The gas response (*S*) to the tested gases was defined as:

*S* = *R*_a_/*R*_g_

where *R*_g_ and *R*_a_ were the resistance in air and in the tested gas, respectively. The response and recovery times were chosen from the times to achieve at least 90% resistance change of the saturated value. The light-driven gas sensing properties of the obtained samples were investigated on the reconstructive WS-30A system (Figure 1). A white LED lamp was used as the light source (CEL−LED100HA, Beijing CEAULIGHT Co.), and the gas-sensing properties were measured under LED illumination. The gas response was calculated by the above method. Photocatalytic performance for RhB degradation was measured and the experimental process was described in detail in the supplementary material (S1).

## 3. Results and discussion

### 3.1. Morphology and Composition of Materials

**Figure 2:**
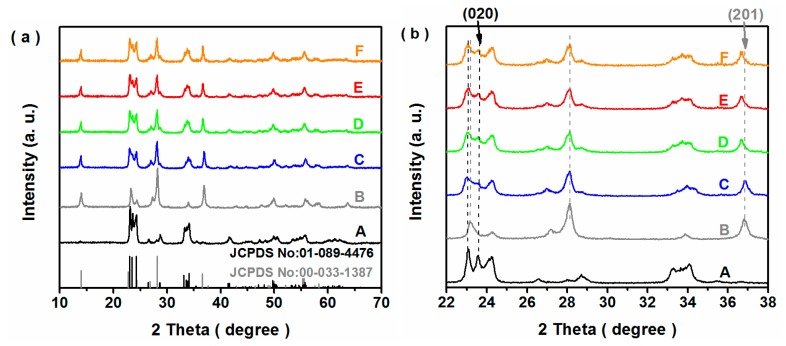
XRD patterns (**a**) and locally enlarged XRD patterns (**b**) of the *m*−WO_3_ (A), *h*−WO_3_ (B), *h/m*−WO_3_ (C), 1.0Fe−*h/m*−WO_3_ (D), 1.25Fe−*h/m*−WO_3_ (E) and 1.5Fe−*h/m*−WO_3_ (F) samples.

The crystal compositions of the as-prepared samples were measured and the results are shown in Figure 2. The main peaks of the *m*−WO_3_ sample were observed at 23.0°, 23.5°, 24.3°, 33.1°, 33.5°, 33.8°, 34.0°, 49.5° and 54.1°, which corresponded to (0 0 2), (0 2 0), (2 0 0), (0 2 2), (−2 0 2), (2 0 2) and (2 2 0) diffraction planes of monoclinic WO_3_ (JCPDS No: 01−089−4476), respectively. For the *h*−WO_3_ sample, the distinct peaks at 13.9°, 22.7°, 24.3°, 28.2° and 36.6° were well in agreement with those of (1 0 0), (0 0 1), (1 1 0), (2 0 0) and (2 0 1) planes of hexagonal WO_3_ (JCPDS No: 00−033−1387). Additionally, both monoclinic and hexagonal phases coexisted in the Fe−doped and undoped *h*/*m*−WO_3_ samples. The locally enlarged XRD patterns were presented in Figure 2b, and it was noteworthy that the characteristic peak of the (2 0 1) crystal plane of hexagonal phase WO_3_ in the Fe-doped *h*/*m*−WO_3_ sample shifted toward the lower angle region. With the increase of Fe content, the peak of (0 2 0) crystal plane of monoclinic-phase WO_3_ also exhibited a slight shift. The above results indicated that the Fe impurity was successfully doped into the crystal structure of WO_3_.

**Figure 3:**
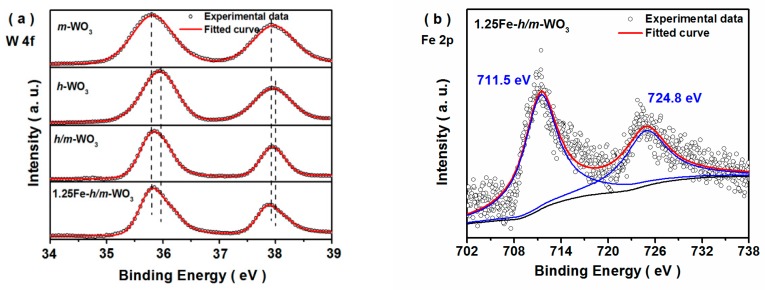
X-ray photoelectron spectroscopy (XPS) spectra of W 2f **(a**) and Fe 2p (**b**) for the as−prepared samples.

The surface compositions of the as−prepared samples were investigated by XPS measurement. As shown in Figure 3, the high-resolution XPS spectra for W 4f and Fe 2p were deconvoluted by the Gaussian−Lorenzian method. Two evident peaks observed for *m*−WO_3_ at the binding energies of 37.9 and 35.8 eV were assigned to W 4f_5/2_ and W 4f_7/2_, respectively, which were in accordance with the reported results of monoclinic WO_3_ [31]. The peaks at 38.0 and 36.0 eV corresponded to the characteristic peaks of W(VI) in hexagonal phase WO_3_ [32,33]. In *h*/*m*−WO_3_ and 1.25Fe−*h*/*m*−WO_3_ samples, the characteristic peaks of W(VI) for monoclinic and hexagonal phase were discovered in the *m*−WO_3_ and *h*−WO_3_, respectively [32,34]. Besides, it was observed that W(VI) 4f_7/2_ and W(V) 4f_5/2_ peaks of *m*−WO_3_ and *h*−WO_3_ shifted slightly compared with those of *h*/*m*−WO_3_ and 1.25Fe−*h*/*m*−WO_3_, which were caused by the formation of phase junctions and Fe-doping [6,15]. In the high-resolution Fe 2p spectrum (Figure 3b), the deconvolution peaks were resolved into four components. In particular, two distinct peaks at binding energies of 711.5 and 724.8 eV corresponded to the characteristic peaks of Fe(III) for 2p_3/2_ and 2p_1/2_, respectively [35]. Combined with the XRD results, Fe impurity was shown to be successfully doped into the *h*/*m*−WO_3_ phase junction.

**Figure 4:**
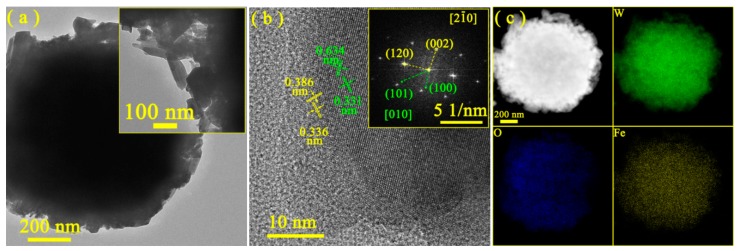
TEM images (**a**,**b**) and EDX mapping (**c**) of the 1.25Fe−*h/m*−WO_3_ sample.

The morphologies of the as−prepared samples were investigated by SEM measurements. As shown in Figure S1, distinct agglomeration was observed for all the samples. The size of agglomerated particles for *m*−WO_3_ and *h*−WO_3_ reached about 1.1 μm. It was noticeable that cuboid nanoparticles appeared in the 1.25Fe−*h*/*m*−WO_3_ sample. Additionally, there was no obvious difference for the surface areas of the samples, based on the result of the N_2_ absorption−desorption measurements (Table S2). Figure 4 displayed the representative TEM images of representative 1.25Fe−*h/m*−WO_3_ sample. As shown in Figure 4a, 1.25Fe−*h/m*−WO_3_ was consisted of cuboid and anomaly nanoparticles with partial agglomeration. The HRTEM image of 1.25Fe−*h/m*−WO_3_ with representative phase junction structure was shown in Figure 4b. The lattice fringes with interplanar spacings of 0.386 and 0.336 nm were assigned to (1 2 0) and (0 0 2) facets of monoclinic WO_3_. Additionally, the lattice fringes of (1 0 1) and (1 0 0) planes for hexagonal WO_3_ were observed, based on the resolved interplanar spacings of 0.631 and 0.331 nm. The simulated FFT pattern is available for locating the highlighted spots, which could be well-indexed to its planes. As shown in the inset of Figure 4b, the zone axis of 1.25Fe−*h/m*−WO_3_ sample was calculated from the cross product of the two perpendicular vectors. The junction plane for monoclinic WO_3_ was thus assigned to (1 2 0) and (0 0 2) facets with the [2 −1 0] zone axis. Similar analysis indicated that the (1 0 1) and (1 0 0) planes of hexagonal WO_3_ existed along with the [0 1 0] axis direction. The results indicated the formation of hexagonal and monoclinic WO_3_ phase junctions. The high-resolution TEM images and FFT pattern, combined with XRD results, revealed both hexagonal phase and monoclinic phase were present and fused at the interfaces of 1.25Fe−*h/m*−WO_3_. To further study the detailed elemental distribution, energy-filtered W, Fe and O maps of 1.25Fe−*h/m*−WO_3_ in Figure 4c revealed that all the elements were uniformly distributed on the particle surface. Based on the above analysis, we conclude the Fe(III) impurity has been doped into the hexagonal and monoclinic WO_3_ phase junction samples.

UV−Vis light absorption spectra were investigated to determine optical properties of the obtained samples (Figure S3). The absorption edges of WO_3_−based samples were around 440 nm, and the absorption abilities of hexagonal WO_3_-based samples were promoted in the range of 550−800 nm, as compared with that of *m*−WO_3_, which may be due to the oxygen vacancies of the hexagonal phase WO_3_ structure. The indirect band gaps (E_g_) of WO_3_-based photocatalysts were calculated by the Kubelka−Munk method [34]. The E_g_ values of *m*−WO_3_ and *h*−WO_3_ reached 2.76 and 2.61 eV, respectively. Moreover, the apparent E_g_ values of *h/m*−WO_3_ and Fe−*h/m*−WO_3_ samples were between 2.48 and 2.56 eV. With the increase of Fe content, the apparent E_g_ values gradually decreased, which implied that Fe doping broadened the light absorption range of WO_3_−based materials.

### 3.2. Performance for Acetone Analysis

**Figure 5:**
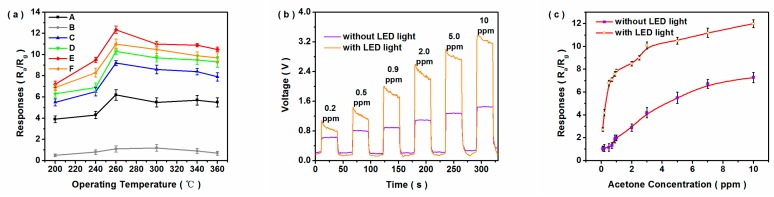
(**a**) Responses of the as-prepared gas sensors to 10 ppm of acetone as a function of operating temperature (A: *m*−WO_3_, B: *h*−WO_3_, C: *h/m*−WO_3_, D: 1.0Fe−*h/m*−WO_3_, E: 1.25Fe−*h/m*−WO_3_, F: 1.5Fe−*h/m*−WO_3_); response–recovery curves (**b**) and corresponding responses (**c**) to acetone gas with concentration ranging from 0.1 to 10 ppm for 1.25Fe−*h/m*−WO_3_ at 90% *RH* atmosphere.

The sensing properties of WO_3_−based sensors were obviously dependent on the operating temperature. As shown in Figure 5a, all the prepared sensors reached maximum responses at 260 °C to 10 ppm acetone. In particular, 1.25Fe−*h/m*−WO_3_ exhibited the optimal response, which was attributed to the formation of phase junction and Fe doping. Moreover, the sensing response of 1.25Fe−*h/m*−WO_3_ to different concentrations of acetone were measured at 260 °C at a low *RH* atmosphere (<20% *RH*). As shown in Figure S3, there were sudden rises and declines of the voltage values when acetone gas was added and discharged, respectively, which indicated that 1.25Fe−*h/m*−WO_3_ possessed fast response/recovery speed towards acetone molecules. Particularly, the response of 1.25Fe−*h/m*−WO_3_ to acetone was linear from 0.1 to 1 ppm, which was not suitable for detection of the relatively high concentration of acetone for exhaled breath analysis under the humid condition. The response of WO_3_-based sensor to acetone was thus investigated in the concentration range of 0.2−10 ppm at 90% *RH* atmosphere. As shown in Figure S4, the responses to acetone decreased at 90% *RH* atmosphere, compared with those at a low *RH* atmosphere (<20% *RH*). Moreover, 1.25Fe−*h/m*−WO_3_ exhibited an enhanced response to acetone at 90% *RH* atmosphere under LED light illumination (Figure 5b,c), and the sensor required more time to establish dynamic balance because more energetic electrons were generated by the light energy. As shown in Figure S5, the response exhibited a linear relation with acetone concentration in the range of 0.5−2.5 ppm at 90% *RH* atmosphere. Additionally, the acetone-sensing properties of other sensors were also improved under LED light illumination (Figure S6). The results indicated that light irradiation improved the responses of Fe-doped *h/m*−WO_3_ gas sensor at high *RH* atmosphere. Hence, Fe-doped *h/m*−WO_3_ phase junction sensors could be used to screen type II diabetes by analysis of acetone in exhaled breath.

**Figure 6:**
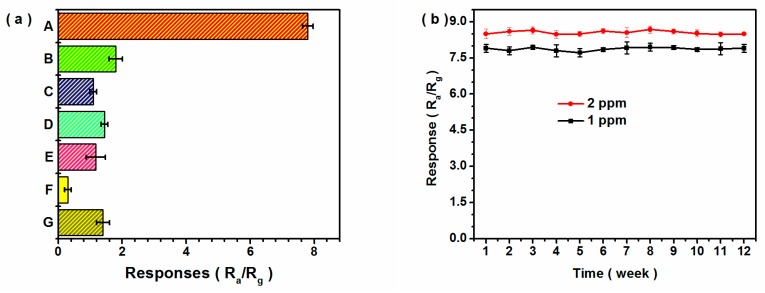
(**a**) Sensing responses of 1.25Fe−*h/m*−WO_3_ sensor to 1 ppm acetone and 1 ppm other gases under LED illumination at 90% *RH* atmosphere (A: acetone, B: ethanol, C: methanol, D: toluene, E: NH_3_, F: NO, G: CO). (**b**) Long−term stability of 1.25Fe−*h/m*−WO_3_ sensor to 1 and 2 ppm acetone over a duration of 3 months under illumination at 90% *RH* atmosphere.

High selectivity for semiconductor sensors is one of the fundamental properties required for breath analysis. Six typical gases including ethanol, methanol, toluene, ammonia (NH_3_), nitric oxide (NO) and carbon monoxide (CO) existed in the exhaled breath. The sensing responses of 1.25Fe−*h/m*−WO_3_ sample to 1 ppm acetone and 1 ppm other gases were shown in Figure 6a. The responses to other gases were less than 1.8 under illumination at 90% *RH* atmosphere. Additionally, the mixtures of 1 ppm acetone and 1 ppm different interference gases were injected in the detection system, and the sensing results (Table S3) indicated that the interference gases such as ethanol, methanol, toluene, NH_3_, NO and CO had no significant effect on the response to acetone. As for the multicomponent gas mixture, the change of response value was less than 7% compared with the original response to single acetone. The results indicated that 1.25Fe−*h/m*−WO_3_ exhibited good selective responses to acetone gas. Apart from selectivity, stability is crucial for practical application of gas sensing materials. The responses of an identical sensor to 1 and 2 ppm acetone were evaluated every week in the duration of three months. As shown in Figure 6b, the response exhibited a slight fluctuation, and it indicated that the 1.25Fe−*h*/*m*−WO_3_ sensor possessed excellent reproducibility and long-term stability.

### 3.3. Mechanism

**Figure 7:**
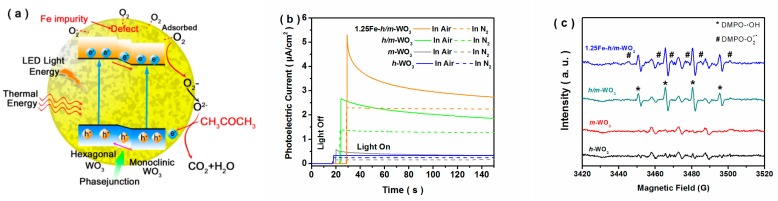
Schematic diagram of the possible gas-sensing mechanism under illumination (**a**), electron spin resonance (ESR) spectra of the obtained samples in water under illumination (**b**), and I−t curves of the obtained samples under different atmospheres (**c**).

In the general sensing mechanism for acetone analysis, free electrons of the WO_3_ sensor at the working temperature were trapped by adsorbed oxygen molecules on the surface, forming reactive oxygen species. Upon exposure to acetone gas, chemisorbed oxygen components reacted with acetone molecules, and the electrons were released back to WO_3_. In this study, Fe-doping resulted in the generation of holes via defect reactions for the 1.25Fe−*h/m*−WO_3_ sensor, promoting the adsorption ability of oxygen molecules [24,25]. In addition, more electrons were generated under LED light illumination (Figure 7a), and inhibited recombination of photo-induced electrons and holes was achieved by the phase junction and Fe doping, which facilitated the formation of reactive oxygen species. Based on the above mechanism, rapid combination of electrons and adsorbed oxygen molecules played a key role for the enhanced acetone sensing performance. Photocurrent decay measurements were further conducted to explore the electron transfer. Under N_2_ atmosphere, photocurrents of all the samples were stabilized during the entire period of irradiation (Figure 7b). However, the photocurrent gradually decayed under air atmosphere. The photocurrents of *h/m*−WO_3_ phase junction samples were higher than those of *h*−WO_3_ and *m*−WO_3_ samples, which implied that the separation of photo-induced electrons and holes was promoted by the phase junction. Notably, the photocurrent of 1.25Fe−*h/m*−WO_3_ decayed faster than those of the undoped WO_3_-based samples. It was noteworthy that the decay of photocurrent was mainly due to the competitive electron trapping between O_2_ and FTO substrate [36]. Additionally, superoxide radicals were important for gas-sensing properties of semiconductors [18], and electron spin resonance (ESR) measurements were further conducted to demonstrate the formation of superoxide radicals. 5,5−Dimethyl−1−pyrroline N−oxide (DMPO) was adopted as a trapping agent for free radicals. As shown in Figure 7c, three peaks were observed for the *h*−WO_3_ and *m*−WO_3_ samples, which were caused by DMPO oxidization. The 1:2:2:1 quartet peaks, representing the characteristic signals of DMPO−**·**OH [37,38], were discovered for the *h/m*−WO_3_ sample. It was notable that the 1:1:1:1:1:1 six−fold peaks of DMPO−O_2_^−^**·** were found for the 1.25Fe−*h*/*m*−WO_3_ sample, which indicated that abundant superoxide radicals were formed due to the interaction of dissolved oxygen and photo-induced electrons [37]. Photocatalytic degradation performances of WO_3_−based samples were further measured to prove the formation of superoxide radicals, and the results were shown in supplementary material S2. Based on the photocatalytic performance (Figure S7–S11), Fe impurity facilitated the photoinduced electron transfer from WO_3_ to the adsorbed oxygen, leading to the generation of superoxide radicals [37,39]. Hence, the formation of WO_3_ phase junctions was favorable for carrier separation, and Fe doping benefited the oxygen adsorption on the WO_3_ surface, leading to increasing electron transfer and promoting the generation of superoxide radicals.

## 4. Conclusions

A series of Fe-doped hexagonal and monoclinic WO_3_ phase junction materials were synthesized by the hydrothermal−calcination method. Under LED illumination, the optimized 1.25Fe−*h/m*−WO_3_ sensor exhibited higher responses to acetone, compared with other as−prepared sensors, and excellent linearity between responses and acetone concentration (0.5−2.5 ppm) was achieved at 90% *RH*. Meanwhile, the 1.25Fe−*h/m*−WO_3_ sensor exhibited good acetone selectivity and stability over three months, which provided an opportunity for the diagnosis of diabetes. Additionally, the Fe-doped WO_3_ phase junction catalyst showed good photocatalytic performance for RhB degradation. It was demonstrated that the formation of WO_3_ phase junctions benefited the carrier separation, and Fe doping was favored oxygen adsorption on the WO_3_ surface, promoting the electron transfer and the generation of superoxide radicals. The results herein may be useful for rational design of gas sensor materials with high selectivity and stability, offering an alternative for noninvasive diagnosis of diabetes. 

## Supplementary Materials

The following are available online at https://www.mdpi.com/2079-4991/10/2/398/s1, S1. Experimental process of photocatalytic degradation. S2. Photocatalytic performance for RhB degradation. Figure S1: SEM images of the *h/m*−WO_3_ (a), 1.0Fe−*h/m*−WO_3_ (b), 1.25Fe−*h/m*−WO_3_ (c) and 1.5Fe−*h/m*−WO_3_ (d) samples. Figure S2: UV−Vis light absorption spectra of the as−prepared WO_3_ samples (A: *m*−WO_3_, B: *h*−WO_3_, C: *h/m*−WO_3_, D: 1.0Fe−*h/m*−WO_3_, E: 1.25Fe−*h/m*−WO_3_, F: 1.5Fe−*h/m*−WO_3_). Figure S3: Response−recovery curves (a) and corresponding responses (b) at low RH atmosphere (< 20%) to acetone gas with the different concentrations (from 0.1 to 10 ppm) for 1.25Fe−*h/m*−WO_3_. Figure S4: Corresponding responses (b) to acetone gas with the different concentrations (from 0.1 to 10 ppm) for 1.25Fe−*h/m*−WO_3_ at different humidity. (Inset: responses vs. acetone concentration from 0.2 to 1 ppm). Figure S5: Corresponding response to acetone gas with the different concentrations from 0.1 to 10 ppm for 1.25Fe−*h/m*−WO_3_ with/without white LED illumination at 90% *RH* atmosphere. Figure S6: Sensing responses of other sensors to 10 ppm acetone under LED illumination at 90 % *RH* atmosphere (*m*−WO_3_ (A), *h*−WO_3_ (B), *h/m*−WO_3_ (C), 1.0Fe−*h/m*−WO_3_ (D), 1.25Fe−*h/m*−WO_3_ (E) and 1.5Fe−*h/m*−WO_3_ (F) samples). Figure S7: Photocatalytic activity (a) and kinetic curves (b) of the as−prepared samples for RhB degradation. Figure S8: Photocatalytic performance for RhB degradation after 40 min illumination in the cycling experiment. Figure S9: XRD pattern of 1.25Fe−*h/m*−WO_3_ before/after cycling. Figure S10: High−resolution XPS spectra for Fe 2p of 1.25Fe−*h/m*−WO_3_ before/after cycling. Figure S11: Photocatalytic activity for RhB degradation in a controlled experiment (A: without scavenger in air atmosphere, B: without scavenger in N_2_ atmosphere, C: addition of EDTA−Na_2_, D: addition of KBrO_3_, and E: addition of 4−benzoquinone). Table S1: Fe/W content ratio of the obtained samples by the ICP−AES measurement. Table S2: Surface area of the obtained samples by the N_2_ absorption−desorption measurement. Table S3: Responses to acetone under different interfering gases for the optimized sensor. Table S4: Pseudo−first order rate constant k of the obtained samples for RhB degradation. Table S5: Fe/W content ratio of the 1.25Fe−*h/m*−WO_3_ samples in cycling by the ICP−AES measurement.
