# Supplementary material for: Enhanced Photo-Assisted Acetone Gas Sensor and Efficient Photocatalytic Degradation Using Fe-Doped Hexagonal and Monoclinic WO3 Phase−Junction
Source: Nanomaterials (Basel). 2020 Feb 24;10(2):398. doi: 10.3390/nano10020398 (PMC7075328; doi:10.3390/nano10020398)
Supplement: Supplementary file 1 [file nanomaterials-10-00398-s001.pdf]

Supplementary Material

Enhanced Photo-Assisted Acetone Gas Sensor and Efficient Photocatalytic Degradation Using Fe Doped Hexagonal and Monoclinic WO_3 Phase-Junction

Ji-Chao Wang [1, 2], Weina Shi [3*], Xue-Qin Sun [1], Fang-Yan Wu [1], Yu Li [4], Yuxia Hou [1*].

[1] College of Chemistry and Chemical Engineering, Henan Institute of Science and Technology, Xinxiang 453000, China.

[2] College of Chemistry and Molecular Engineering, Zhengzhou University, Zhengzhou 450052, China.

[3] College of Chemistry and Chemical Engineering, Xinxiang University, Xinxiang 453000, China.

[4] College of Chemical Engineering and Materials, Zhengzhou University of Light Industry, Zhengzhou, 450000, China

*Corresponding authors:

Dr. W. Shi (E-mail: shiweina516@163.com)

Dr. Y. Hou (E-mail: yxhou@163.com)

S1. Experimental process of photocatalytic degradation

A 300 W Xe arc lamp (CEL-HXF300, Beijing CEAULIGHT Co. China) with an ultraviolet cutoff filter ($\lambda \geq 400$ nm) was employed as the visible-light source. 50 ± 3 mg photocatalyst was firstly dispersed into 100 mL of 20 mg/L RhB aqueous solution. Before irradiation, the solution was strongly stirred in the dark for 90 min, confirming adsorption-desorption equilibrium between photocatalyst and pollutant molecules. In the photocatalytic process, the solution temperature should be kept at about 3 ℃, avoiding the effect of heat treatment on the degradation. About 3 mL of the fluid solution was taken out of 10 min intervals. The photocatalyst should be separated through 8000 rpm centrifugation. The RhB concentration was measured by the UV-Visible spectrophotometer with the work wavelength of 554 nm.

S2. Photocatalytic performance for RhB degradation

The photocatalytic activity for RhB degradation was studied under visible-light illumination. In Figure S7a, the degradation efficiency of *h*/*m*-WO_3 was higher than that of single *m*-WO_3 and *h*-WO_3, which was caused by effective separation of photoinduced electron-hole pairs in phase-junction. The photocatalytic activities of the Fe doped *h*/*m*-WO_3 samples were further promoted, and the degradation ratio of 1.25Fe-*h*/*m*-WO_3 particularly reached above 92 % after 40 min illumination. To further investigate the reaction kinetics, the equation S1 was adopted to build the pseudo-first order model for RhB degradation process.

$$\ln(c_0/c) = -kt \quad \text{(S1)}$$

where c_0 and c were on behalf of the RhB concentrations at stating and time t, respectively, and k was corresponding on the pseudo-first order rate constant. The relevant rate constant k was got by the above method and summarized in Table S4. The correlation coefficient (R) for pseudo-first-order kinetics could reach above 0.99 in

RhB degradation (Figure S7b). The rate constant distinctly increased with the existence of Fe doping. The *k* value of 1.25Fe-*h/m*-WO_3 sample for RhB degradation was 0.06876, which was approximately 31, 9.4 and 4.8 times of the single *h*-WO_3, *m*-WO_3 and undoped *h/m*-WO_3 samples, respectively. Due to Fe doping, Photocatalytic activities of the Fe doped *h/m*-WO_3 samples were promoted.

Catalytic cycling performance of 1.25Fe-*h/m*-WO_3 for RhB degradation was studied. In Figure S8, the photocatalytic activity for degradation decreased gently, and the degradation efficiency remained above 85 % in the 8th cycling under visible light illumination. Combined with the XRD and XPS results after photocatalysis (Figure S9 and S10), the crystal structure and surface chemical composition of 1.25Fe-*h/m*-WO_3 after illumination did not change significantly, compared with that before illumination. Moreover, on the basis of the ICP-AES results (Table S5), the content of doped Fe distinctly decreased after 8th cycling, which possibly caused the decline of photocatalytic activity.

According to result of controlled experiments (Figure S11), the photocatalytic activity for RhB degradation obviously decreased at N_2 atmosphere, which suggested that dissolved oxygen was crucial for RhB degradation. On the contrary, the photocatalytic activity increased with the addition of EDTA-Na_2 (hole scavenger), which implied that photo-induced electrons were the main role for degradation activity. Additionally, the photocatalytic activity disappeared in the presence of $KBrO_3$ (electron scavenger) and 4-benzoquinone (4-BQ, superoxide radical scavenger). Hence, superoxide free radical was absolutely critical for photocatalytic RhB degradation.

Table S1. Fe/W content ratio of the obtained samples by the ICP-AES measurement.

Sample	Nominal	ICP-AES
m-WO_3	0:100	0:100
h-WO_3	0:100	0:100
h/m-WO_3	0:100	0:100
1.0Fe-*h/m*-WO_3	1.0:100	0.59:100
1.25Fe-*h/m*-WO_3	1.25:100	0.72:100
1.25Fe-*h/m*-WO_3-H	1.25:100	0.68:100
1.5Fe-*h/m*-WO_3	1.5:100	0.85:100

Table S2. Surface area of the obtained samples by the N_2 absorption-desorption measurement.

Sample	Surface area (m^2/g)	Sample	Surface area (m^2/g)
m-WO_3	35.2	1.0Fe-*h/m*-WO_3	37.5
h-WO_3	36.9	1.25Fe-*h/m*-WO_3	41.0
h/m-WO_3	38.0	1.5Fe-*h/m*-WO_3	45.8

Table S3. Responses to acetone under different interfering gases for the optimized sensor.

**Gases**	**Response***
1 ppm acetone	7.8

1 ppm acetone+ 1 ppm ethanol	8.1
1 ppm acetone+ 1 ppm methanol	7.9
1 ppm acetone+ 1 ppm toluene	7.8
1 ppm acetone+ 1 ppm ammonia	7.9
1 ppm acetone+ 1 ppm nitric oxide	7.7
1 ppm acetone+ 1 ppm carbon monoxide	7.9
1 ppm acetone+ 1 ppm ethanol+ 1 ppm toluene+ 1 ppm ammonia+ 1 ppm nitric oxide+ 1 ppm carbon monoxide	8.1
2 ppm acetone	8.5
2 ppm acetone+ 1 ppm ethanol+ 1 ppm toluene+ 1 ppm ammonia+ 1 ppm nitric oxide+ 1 ppm carbon monoxide	8.8

* the value response of sample was the average value for response of six parallel gas sensors.

Table S4. Pseudo-first order rate constant *k* of the obtained samples for RhB degradation.

Sample	*k* ($\times 10^{-2}$ min^{-1})	Sample	*k* ($\times 10^{-2}$ min^{-1})
m-WO_3	0.729	1.0Fe-*h*/*m*-WO_3	4.501
h-WO_3	0.217	1.25Fe-*h*/*m*-WO_3	6.876
h/*m*-WO_3	1.422	1.5Fe-*h*/*m*-WO_3	3.222

Table S5. Fe/W content ratio of the 1.25Fe-*h*/*m*-WO_3 samples in cycling by the ICP-AES measurement.

Cycling Number	ICP-AES

0	0.72:100
1	0.69:100
3	0.70:100
5	0.65:100
8	0.60:100
3 months*	0.70:100

* the 1.25Fe-*h/m*-WO_3 sample was got after 3 months sensing measurement.

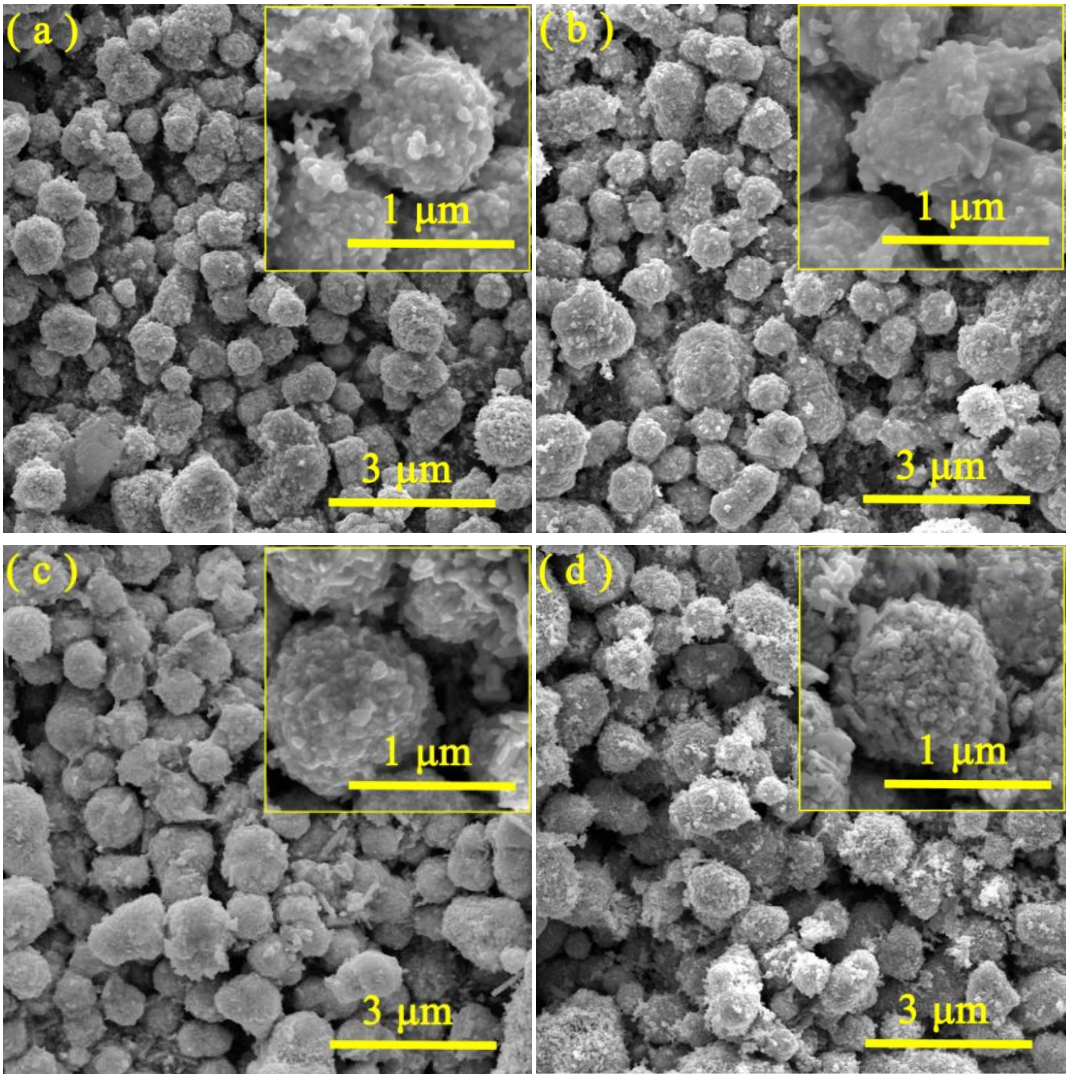

Figure S1. SEM images of the *h/m*-WO_3 (a), 1.0Fe-*h/m*-WO_3 (b), 1.25Fe-*h/m*-WO_3 (c) and 1.5Fe-*h/m*-WO_3 (d) samples.

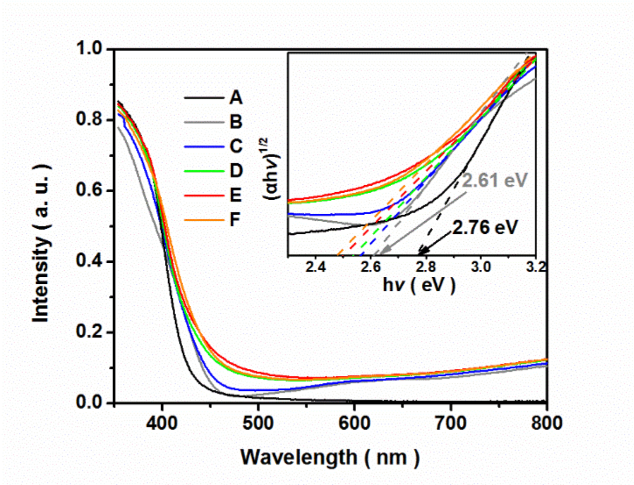

Figure S2. UV-Vis light absorption spectra of the as-prepared WO_3 samples (A: *m*-WO_3, B: *h*-WO_3, C: *h/m*-WO_3, D: 1.0Fe-*h/m*-WO_3, E: 1.25Fe-h/m-WO_3, F: 1.5Fe-*h/m*-WO_3).

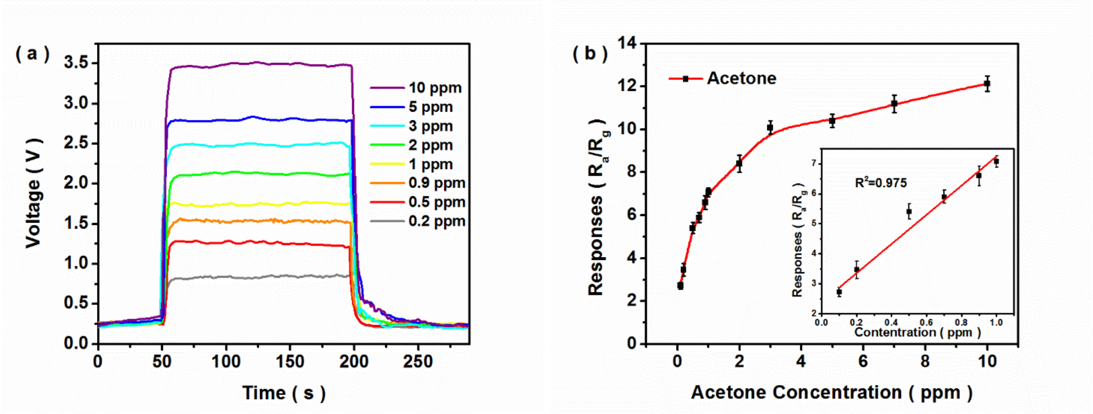

Figure S3. Response-recovery curves (a) and corresponding responses (b) at low *RH* atmosphere (< 20%) to acetone gas with the different concentration (from 0.1 to 10 ppm) for 1.25Fe-*h/m*-WO_3.

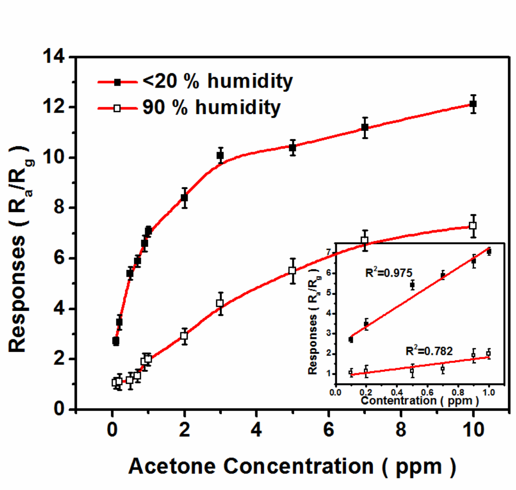

Figure S4. Corresponding responses (b) to acetone gas with the different concentrations (from 0.1 to 10 ppm) for 1.25Fe-*h/m*-WO_3 at different humidity atmosphere. (Inset: responses *vs*. acetone-concentration from 0.2 to 1 ppm).

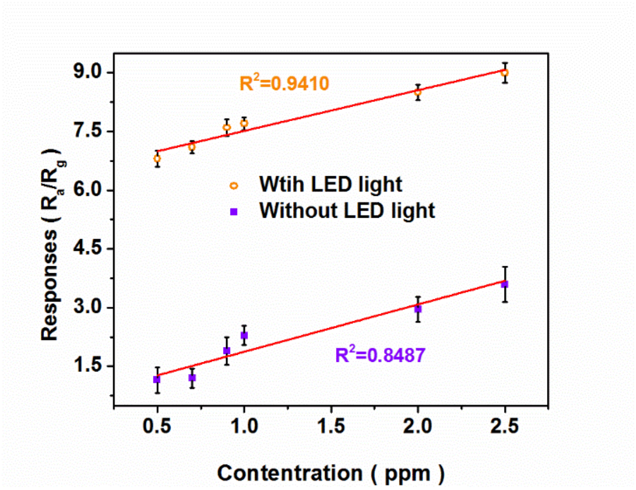

Figure S5. Corresponding response to acetone gas with the different concentration from 0.5 to 2.5 ppm for 1.25Fe-*h*/*m*-WO_3 with/without white LED illumination at 90 % *RH* atmosphere.

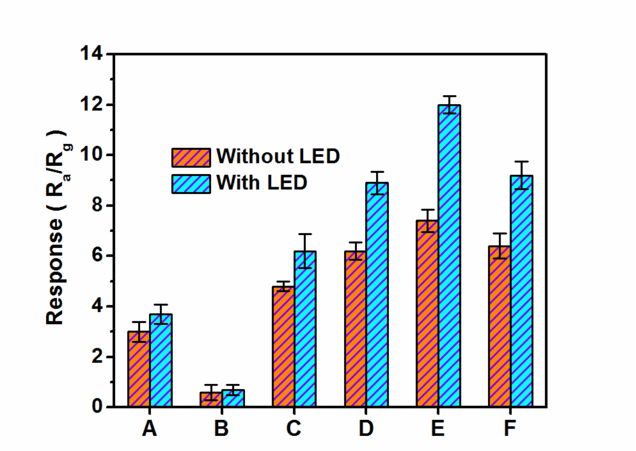

Figure S6. Sensing responses of other sensors to 10 ppm acetone under LED illumination at 90 % *RH* atmosphere (m-WO_3 (A), *h*-WO_3 (B), *h*/*m*-WO_3 (C), 1.0Fe-*h*/*m*-WO_3 (D), 1.25Fe-*h*/*m*-WO_3 (E) and 1.5Fe-*h*/*m*-WO_3 (F) samples)

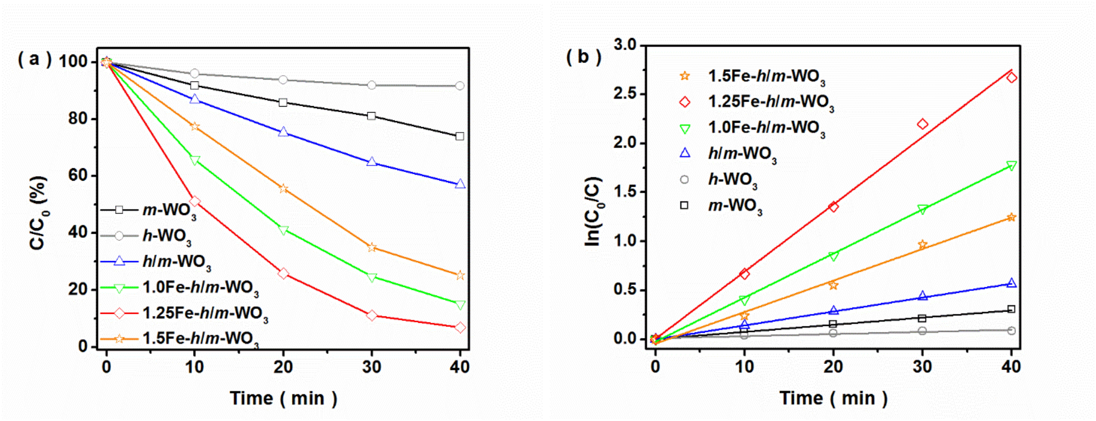

Figure S7. Photocatalytic activity (a) and kinetic curves (b) of the as-prepared samples for RhB degradation.

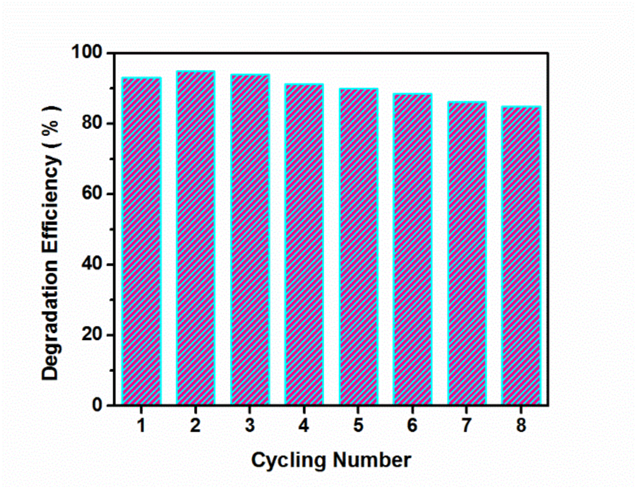

Figure S8. Photocatalytic performance for RhB degradation after 40 min illumination in cycling experiment.

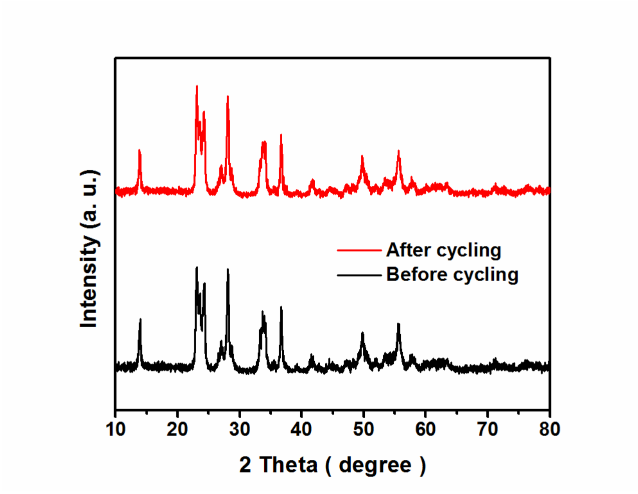

Figure S9. XRD pattern of 1.25Fe-*h/m*-WO_3 before/after cycling.

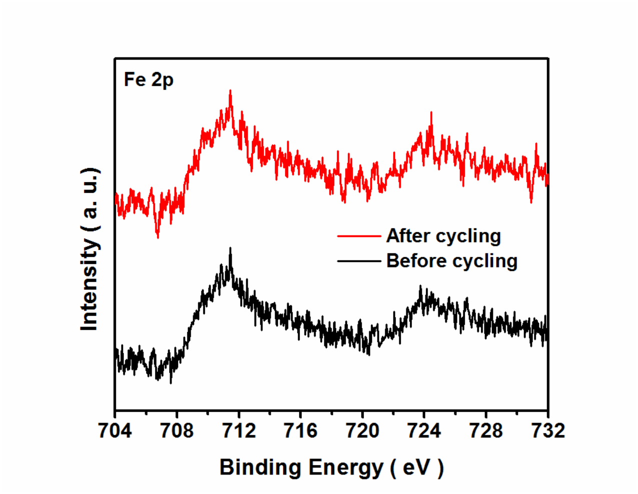

Figure S10. High-resolution XPS spectra for Fe 2p of 1.25Fe-*h/m*-WO_3 before/after cycling.

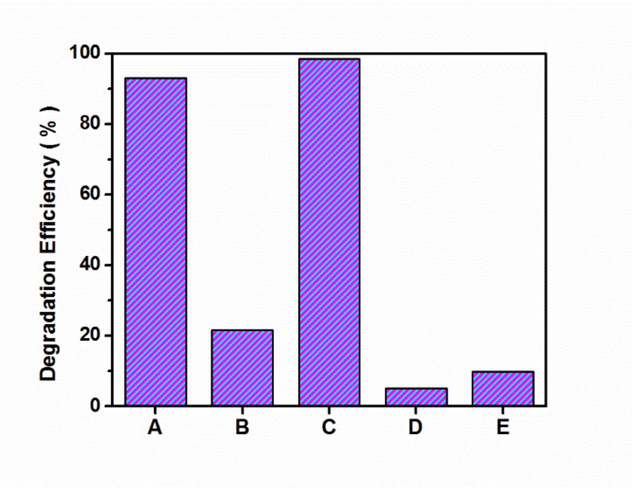

Figure S11. Photocatalytic activity for RhB degradation in controlled experiment (A: without scavenger in air atmosphere, B: without scavenger in N_2 atmosphere, C: addition of EDTA-Na_2, D: addition of $KBrO_3$, and E: addition of 4-benzoquinone.).
